# Effectiveness, barriers, and facilitators of interventions delivered by traditional healers for the treatment of common mental disorders: A systematic review

**DOI:** 10.1177/13634615241273001

**Published:** 2024-09-24

**Authors:** Mujeeb Masud Bhatti, Najma Siddiqi, Hannah Jennings, Saima Afaq, Aatik Arsh, Bilal Ahmed Khan

**Affiliations:** 1 Department of Health Sciences, 8748 University of York, York, UK; 2 Institute of Public Health and Social Sciences, 194863 Khyber Medical University, Peshawar, Pakistan

**Keywords:** barriers, effectiveness, facilitators, traditional healers

## Abstract

We investigated the effectiveness of interventions provided by traditional healers for common mental disorders (CMDs) together with associated barriers and facilitators. Electronic databases including MEDLINE, APA Psych Info, Allied and Complementary Medicine, Embase, CINAHL, Social Science Citation Index, and Scopus were searched from inception until October 2021. Randomised controlled trials (RCTs) assessing interventions by traditional healers for CMDs and qualitative and mixed-methods studies examining traditional healers and their attendees’ views about the treatment of CMDs by traditional healers were included. Cochrane Risk of Bias Assessment tool (RoB-1) and Critical Appraisal Skills Programme (CASP) were used for the quality assessment of studies. A meta-analysis and thematic synthesis were conducted. Sixteen RCTs (1,132 participants) and 17 qualitative or mixed-methods studies (380 participants) were included. Improvement in symptoms was greater for interventions by traditional healers compared to control groups for both depression and anxiety. Subgroup analyses indicated that only “spiritual passe” interventions showed improvement in depression and anxiety, and participants with co-morbid anxiety and physical conditions showed improvement in anxiety. Facilitators to engaging with interventions by traditional healers were shared faith-based worldview exhibited by traditional healers and their attendees and perceived effectiveness of traditional healing. Stigma and concealing mental illness were found to be barriers not only to formal healthcare but also to traditional healers’ services. Interventions by traditional healers such as “spiritual passe” are effective in improving CMDs. However, evidence is still limited due to the low quality of studies and lack of long-term evidence.

## Introduction

Common mental disorders (CMDs), including depressive and anxiety disorders, are highly prevalent. Approximately 600 million people worldwide suffer from depression and a range of anxiety-related disorders (World Health Organization, 2017). Depression is the leading cause of disability, and anxiety disorders are the sixth leading cause of disability worldwide (World Health Organization, 2017). Despite the high burden of mental illness, many affected people do not have access to evidence-based treatment in low- and middle-income countries (LMICs) (World Health Organization, 2009). Financial, infrastructural, and human resources required to reduce the disease burden associated with mental illness are considerably limited (World Health Organization, 2009). Given the lack of mental health services, people may prefer traditional treatments which are embedded in a cultural milieu (Cohen et al., 2021). Growing evidence suggests that a significant proportion of people in LMICs visit traditional healers before they visit formal healthcare professionals (Burns & Tomita, 2015; Khan et al., 2019; Nuri et al., 2018). Collaboration between formal healthcare professionals and traditional healers may have the potential to improve access to mental health care (Musyimi, Henderson et al., 2017; Musyimi, Unanue et al., 2017). A recent study found collaborative psychosocial interventions provided by traditional healers and formal healthcare professionals are feasible and beneficial for the improvement of psychosis symptoms (Gureje et al., 2020). Another study found training and delivery of non-specialised psychotherapy for depression by traditional healers to be feasible (Musyimi, Henderson et al., 2017).

With the increasing availability of primary data and research on traditional healers in mental healthcare, there have been recent efforts to synthesise these data, aiming to draw more robust conclusions. A systematic review focusing on the initial contact with traditional healers in Africa revealed that approximately 17% of individuals with mental illnesses first contact traditional healers, exceeding the 13% who first seek formal mental health services (Burns & Tomita, 2015). Given this reality, there have been some efforts to understand the perspectives of both traditional healers and healthcare professionals regarding potential collaboration. A qualitative review including data from seven (mostly African) countries suggests that, despite differences in their approaches to addressing mental illnesses, both traditional healers and healthcare professionals express a willingness to cooperate (Green & Colucci, 2020). However, healthcare professionals have consistently expressed concerns about the safety and autonomy of individuals who seek traditional healing, while traditional healers often maintain scepticism about the benefits of formal healthcare practices. The effectiveness of interventions delivered by traditional healers has been a long-standing question. Two systematic reviews have been conducted aiming to explore the effects of traditional healing on mental health. Both suggested potential benefits for CMDs while also indicating that people value traditional healing particularly when it comes to mental health (Nortje et al., 2016; Van der Watt et al., 2018).

One of these reviews based on 32 studies (mostly non-randomised and observational) from 20 countries reported that interventions delivered by traditional healers comprise a wide range of practices, including sacred rituals, divination, and prayers (Nortje et al., 2016). This review found that traditional healers’ interventions are beneficial for CMDs but not for severe mental illnesses such as bipolar disorder or psychosis (Nortje et al., 2016). However, the review included observational studies and there were some difficulties with using standardised and uniform methods to extract, synthesise, and critically appraise included studies, precluding drawing conclusions about effectiveness. The second review included qualitative studies exploring the perceived effectiveness of interventions delivered by traditional healers; it reported that individuals with mental illness value such interventions and perceive them to be useful in the alleviation of mental health problems regardless of the availability of alternative formal healthcare services (Van der Watt et al., 2018). Traditional healers’ practices, however, were also considered ineffective in rare cases where ‘a curse was too strong’ compared to the power of the healing method (Van der Watt et al., 2018). There was limited information on barriers and facilitators people may face in accessing and engaging in interventions by traditional healers (Green & Colucci, 2020; Van der Watt et al., 2018). A recent scoping review including studies based in Nepal reported that religious-magical explanations and practices, informal cognitive restructuring methods, and catharsis were common interventions used by traditional healers, but this review did not address effectiveness (Pham et al., 2021).

### Rationale

Randomised controlled trials (RCTs) are the gold standard for evaluating the effectiveness of interventions, and rigorously conducted systematic reviews of RCTs provide the most robust evidence for establishing or revising treatment policies (Evans, 2003). The effectiveness and uptake of any intervention aiming to improve health outcomes are likely to be influenced by barriers and facilitators in a particular service delivery context (Oliver et al., 2014). Understanding barriers and facilitators associated with interventions provided by traditional healers is especially important when current evidence is suggesting potential collaboration between traditional healers and healthcare professionals could be beneficial (Gureje et al., 2020). Understanding such determinants can facilitate in efficient implementation of such programmes (Le et al., 2022). We therefore aimed to explore the effectiveness of interventions for CMDs delivered by traditional healers by synthesising data from RCTs and using standardised methods for synthesis (i.e., meta-analysis) and critical appraisal (Cumpston et al., 2019). We prioritised CMDs due to their high prevalence, and the consideration that treatment of severe mental illnesses typically requires specialist mental health care. Our focus on CMDs is supported by previous reviews indicating the beneficial effects of traditional healing on CMDs only (Nortje et al., 2016; Van der Watt et al., 2018).

Barriers to accessing and engaging with interventions provided by traditional healers are critical to understand, yet have received limited attention. A review examining the perceptions of traditional healers and healthcare professionals regarding their collaboration provided some insights into barriers to collaboration (Green & Colucci, 2020). However, this review did not encompass the perspectives of individuals seeking traditional healing (Green & Colucci, 2020). We, therefore, sought to examine the barriers and facilitators to accessing and engaging with interventions delivered by traditional healers by reviewing qualitative studies based on both traditional healers and their attendees’ perspectives and experiences. This approach enabled us to consider data from a unique perspective, i.e., a barrier to formal healthcare intervention could serve as a facilitator for interventions delivered by traditional healers, and vice versa. To ensure comprehensiveness, we aimed to include interventions provided independently by traditional healers, and also interventions provided by traditional healers in collaboration with formal healthcare providers. We have used the Preferred Reporting Items for Reviews and Meta-Analyses (PRISMA) statement for presenting and reporting this review.

### Review questions

This review seeks to answer the following questions:

1. Are interventions delivered by traditional healers for the management of CMDs effective?
2. What are the perspectives of traditional healers and individuals with CMDs regarding the barriers and facilitators to engaging with and accessing traditional healing for the management of CMD?

## Methods

### Eligibility criteria

Studies fulfilling the following criteria were included in the review:

*Study designs*: RCTs investigating the effectiveness of treatment provided by traditional healers, qualitative studies, and mixed-methods studies.

*Participants*: Individuals with CMD symptoms (self-reported or clinically diagnosed) seeking care from traditional healers and traditional healers expressing their perspectives on their treatment for CMDs. We have used the definition of traditional healers that has been consistently used by previous reviews (Nortje et al., 2016; Van der Watt et al., 2018). Traditional healers were defined as “healers who explicitly appeal to spiritual, magical, or religious explanations for disease and distress” (Nortje et al., 2016, p. 155). We adopted the definition of CMDs as outlined in a commissioned report by the National Centre for Mental Health in the United Kingdom (UK) when formulating clinical guidelines for the National Health Service (NHS) in the UK. CMDs encompass the symptoms associated with major depressive disorder (MDD), anxiety disorders, panic disorder, obsessive compulsive disorder (OCD), and post-traumatic stress disorder (PTSD) as defined by the *Diagnostic and Statistical Manual* (DSM), the International Classification of Diseases (ICD), or as assessed through a standardised evaluation tool (National Collaborating Centre for Mental Health, 2011).

*Intervention*: The intervention includes: any traditional or faith-based intervention provided by traditional healers independently, any evidence-based treatment where traditional healers were trained by healthcare professionals, or any care provided by both traditional and healthcare professionals in collaboration. Studies were excluded if the intervention was limited to traditional healers only providing oral, topical, or inhaled herbal or chemical substances.

*Comparison* (for RCTs): Three types of comparators were considered: 1) usual/routine care: psychological care provided in primary, secondary, or tertiary care routinely; 2) placebo/mock/sham healing 3) control group: no intervention.

*Outcome and phenomenon of interest*: Primary outcomes included any standardised instrument to quantitatively measure depression, anxiety disorders, panic disorder, OCD, and PTSD. No restrictions on the timings of the outcome were placed. The barriers and facilitators relating to the accessibility and engagement with treatment for CMDs by traditional healers were based on reflections and experiences of individuals with CMDs treated by traditional healers, traditional healers’ experiences of treating people with CMDs, and traditional healers’ perspectives and views regarding CMDs.

### Information sources

To ensure comprehensiveness andrelevance, searching more than one bibliographic database is recommended (Lefebvre et al., 2019). We searched seven databases. Databases searched were MEDLINE (Epub Ahead of Print, MEDLINE In-Process & Other Non-Indexed Citations; Ovid interface; 1946 onwards); APA PsychInfo (Ovid interface; 1987 onwards); Allied and Complementary Medicine (AMED Ovid interface; 1985 onwards); Embase (Ovid interface; 1974 onwards); CINAHL (EBSCO Host); Social Science Citation Index (Web of Science, 1956 onwards), and Scopus (1946 onwards). We searched all databases from their inception to October 2021. The reference lists of articles included in recent relevant systematic reviews (Nortje et al., 2016) were also searched. Hand-searching was carried out to find relevant studies in journals, conference proceedings, and unpublished theses.

### Search strategy

The search strategy was developed with the help of an information specialist, and all searches were run in October 2021 by the first author (MMB) (Lefebvre et al., 2019). The search terms include synonyms (including singulars, plurals, and alternative spellings) for two concepts (Traditional healers and Common mental disorders). A mixed-method filter was used. A search filter is a ready-made search strategy, which is efficient in retrieving relevant records and has been tested previously (see the section on Search filters (Lefebvre et al., 2019)). In our case, we used a mixed-methods search filter to retrieve both qualitative and quantitative records given the scope of our study. Search terms included: 1) “Traditional” or “Spiritual”, for example; 2) “Depression” or “Anxiety”, for example; 3) Mixed-methods filter (Appendix B) (Sherif et al., 2016).

### Selection process

Using pre-specified screening criteria, one reviewer (MMB) examined all titles and abstracts to identify eligible papers (Appendix A). At least two reviewers (MMB, AA, and BAK) independently read the full-text articles of all potentially eligible studies to decide which should be included. Queries were discussed between reviewers at each stage, and disagreements were resolved through consensus or third-reviewer arbitration.

### Data items

The Cochrane Collaboration data extraction form for RCTs was used for RCT studies. The form included bibliographic information, study design, population, intervention, comparator, and outcomes (Li et al., 2019). For the qualitative and mixed-methods studies, details including study characteristics, methodology, data collection method, participants, data analysis, and results were extracted. We also carefully extracted verbatim statements of traditional healers and their visitors reported in the included records, which were further used for the synthesis. We used a data extraction form for qualitative studies developed by the first author (MMB) and this was piloted with the first three studies.

### Risk of bias

The Cochrane Collaboration tool for assessing the Risk of Bias, version 1 (RoB-1) was used for critical appraisal of RCTs (Higgins et al., 2011) and the quality of qualitative studies and mixed-method studies was assessed using the Critical Appraisal Skills Program (CASP) checklist (CASP, 2013). The CASP was chosen due to its extensive usage and endorsement by the Cochrane Qualitative and Implementation Methods Group (Long et al., 2020). It is a convenient and user-friendly tool, offering adaptability to accommodate various philosophical foundations that underlie different qualitative studies included in the review (Long et al., 2020). It also emphasises the researcher–participant relationship, allowing evaluation of potential issues related to reflexivity in included studies. Each of the domains or items for risk of bias was rated for each study and further global ratings collating all domains for each study were reported (Higgins et al., 2019).

### Data collection process

The data extraction tools were piloted with the first three studies and were adapted accordingly. Study characteristics (sample and intervention characteristics) were extracted by a single reviewer (MMB), and data related to the outcomes, verbatim statements, and risk of bias assessment were extracted or rated by at least two reviewers (MMB, AA, and BAK) independently, with disagreements being discussed between two reviewers. In case an agreement was not achieved between two reviewers, a third senior reviewer made the final decision.

### Synthesis methods

We narratively summarised the characteristics of participants, interventions, comparisons, and outcomes or phenomena of interest for all studies included in both tables and texts (McKenzie et al., 2019). Quantitative analysis was performed using RevMan 5.4.1. A meta-analysis using the random effect model (Mantel-Haenszel method) was conducted to account for heterogeneity (Field & Gillett, 2010). All studies were based on continuous data, the mean along with the standard deviation was extracted, and the pooled effect was estimated in standardised mean difference (95% confidence interval). Statistical heterogeneity was estimated through the chi-square test (χ^2^) and I^2^ statistics.

Meta-analysis was performed on an “all-time outcome” basis ranging from 3 days to a 6-month outcome. Given the high level of heterogeneity, subgroup analysis based on intervention and participant characteristics was carried out and publication bias was assessed through funnel plot. Studies reporting median and interquartile ranges were included in the meta-analysis, therefore sensitivity analysis was performed by removing those studies to observe its effects on pooled estimates (Deeks et al., 2019).

Thematic synthesis of qualitative findings was used to explore the barriers and facilitators to accessing and engaging with interventions for CMDs by traditional healers (Thomas & Harden, 2008). NVivo was used to organise and manage the qualitative data. All the qualitative or mixed-methods studies were uploaded onto NVivo. Synthesis was performed on the verbatim statements by traditional healers and their attendees reported in studies. The first author, MMB, then followed the process of reading and familiarising himself with the verbatim statements and coding them line by line. The emergent coding approach was followed, the reviewer immersed himself in the data, and coding emerged out of the data rather than pre-determined codes. Similar codes were collated to form subordinate themes. Themes were subsequently categorised under barriers and facilitators. The identified themes along with quotes were described in narration in the results section. Any discrepancies relating to coding and themes were resolved through discussion between two reviewers (MMB and HJ).

## Results

### Study selection

A total of 33 studies were included in the review; 16 studies were clinical trials, and 17 studies were qualitative or mixed methods (Appendix C). Studies that were excluded were: 1) the wrong study design (N = 19); 2) the wrong outcome (N = 10); or 3) the wrong intervention (N = 7) (Appendix C).

### Effectiveness of interventions delivered by traditional healers

#### Study characteristics

A total of 16 studies were selected. Eight were based in High-Income Countries (HICs) (four in the USA, three in the UK, and one in Finland), seven in Higher Middle-Income Countries (HMICs) (six in Brazil and one in Iran), and one in an LMIC (India). All but three studies (church = 1, healing centre = 1, and community setting = 1) were conducted in healthcare settings (Table 1). Depression and anxiety were the only reported outcomes. Nine out of the 16 studies primarily focused on physical conditions along with symptoms of depression and/or anxiety, whilst seven studies primarily addressed depression and/or anxiety (Table 2). Eight studies used a “spiritual passe” intervention, in which healers move their hands above an individual’s body without touching them. Two studies included reiki and energy healing techniques, which focus on curing through the universal energy systems. The remaining five studies used various healing techniques including prayers, building a relationship with God, psychosocial support from lay pastors, and energy healing prana healing. All the trials evaluated interventions provided independently by traditional healers and we did not find any trial focusing on jointly or collaboratively delivered interventions. Ten studies included a mock/sham healing comparison group with an actor (a non-healer volunteer) copying healer actions as a control. The remaining studies did not provide any intervention to the control group (Table 2).

**Table 1.** Included studies.

Study ID	Document type	Country	Study design	Study settings	Quality
(Abbot et al., 2001)	Journal Article	England	RCT	Healthcare Settings	Low
(Boelens et al., 2009)	Journal Article	USA	RCT	Healthcare Settings	Low
(Carneiro, Tosta et al., 2020)	Journal Article	Brazil	RCT	Healthcare Settings	Low
(Carneiro, Oliveira et al., 2020)	Journal Article	Brazil	RCT	Healthcare Settings	Moderate
(Carneiro et al., 2017)	Journal Article	Brazil	RCT	Healthcare Settings	Low
(Cleland et al., 2006)	Journal Article	Scotland	RCT	Healthcare Settings	Low
(Cavalcante et al., 2016)	Journal Article	Brazil	RCT	Healthcare Settings	Moderate
(Gerard et al., 2003)	Journal Article	Scotland	RCT	Healthcare Settings	Very Low
(Jain, 2009)	Thesis/Dissertation	USA	RCT	Healthcare Settings	Low
(Miranda et al., 2020)	Journal Article	Brazil	RCT	Healthcare Settings	Low
(Nikfarjam et al., 2018)	Journal Article	Iran	RCT	Healthcare Settings	Very Low
(Palmer, 1998)	Thesis/Dissertation	USA	RCT	Church	Very Low
(Rajagopal et al., 2018)	Journal Article	India	RCT	Healthcare Settings	Low
(Shore, 2002)	Thesis/Dissertation	USA	RCT	Healing Centre	Low
(Sundblom et al., 1994)	Journal Article	Finland	RC	Healthcare Settings	Low
(dos Santos et al., 2021)	Journal Article	Brazil	RCT	Community Settings	Moderate
(Stansbury et al., 2009)	Journal Article	USA	Qualitative	Community Settings	Poor
(Bryant et al., 2014)	Journal Article	USA	Qualitative	Community Settings	Poor
(Hankerson et al., 2021)	Journal Article	USA	Qualitative	Healthcare Settings	Poor
(Jang et al., 2017)	Journal Article	USA	Qualitative	Church and Community Settings	Moderate
(Andren & McKibbin, 2018)	Journal Article	USA	Mixed Method	Community Settings	Poor
(Kpobi & Swartz, 2018)	Journal Article	Ghana	Qualitative	NA	Moderate
(Lucchetti et al., 2015)	Journal Article	Brazil	Mixed Method	Spiritist/ Healing Centre	Poor
(Moodley et al., 2018)	Journal Article	South Africa	Qualitative	Community Settings	Good
(Payne & Hays, 2016)	Journal Article	USA	Qualitative	Data was collected through social networking utility	Moderate
(Pullen et al., 2021)	Journal Article	Liberia	Qualitative	NA	Good
(Pyne et al., 2021)	Journal Article	USA	Mixed Method	Healthcare Settings	Moderate
(Pyne et al., 2019)	Journal Article	USA	Qualitative	Healthcare Settings	Moderate
(Stansbury, 2011)	Journal Article	USA	Qualitative	NA	Good
(Storck et al., 2000)	Journal Article	USA	Qualitative	Community Settings	Poor
(Tobah, 2017)	Journal Article	Canada	Qualitative	Community Settings	Moderate
(Wahbeh et al., 2017)	Journal Article	USA	Mixed Method	Healthcare Settings	Poor
(Zoellner et al., 2021)	Journal Article	Somalia	Mixed Method	Mosques	Moderate

*Note.* NA = Not Available or not reported in source; RCT = randomised control trial; USA = United States of America.

**Table 2.** Participants, intervention, comparison(s), and outcome(s) in included clinical trials.

Study	Participants	N	Age M (SD)	Females N (%)	Intervention	Comparison (s)	Outcome	CMD measures	Findings
(Abbot et al., 2001)	Chronic Pain	132	52.85 (NA)	59 (56.1)	Face-to-Face Spiritual Healing	Placebo Healing Distant Healing Control	Week 8	HADS	Depression: ND (For all comparisons) Anxiety: ND (For all comparisons)
(Boelens et al., 2009)	Major Depressive Disorder with Anxiety	74	43.85 (13.25)	60 (95.3)	Prayer Intervention	Control	Week 6	HAMS D, HAMS A	Depression: + Anxiety: +
(Carneiro, Tosta et al., 2020)	Surgical risk and systemic alterations with anxiety	59	57 (13.24)	20 (45.5)	Spiritual Passe	Placebo / Mock Healing Standard Medical Care	Day 3	HADS	Anxiety: + (Compared to Placebo and Standard Medical Care)
(Carneiro, Oliveira et al., 2020)	Anxiety and stress among hospital employees	84	38.39 (10.04)	73 (87)	Spiritual Passe	Control	Day 1	DASS	Depression: ND Anxiety: ND
(Carneiro et al., 2017)	Cardiovascular inpatients	48	58.33 (17.1)	18 (43.9)	Spiritist Passe	Placebo / Mock Healing Control	Day 3	HADS	Depression: + (Compared to both Placebo and Control) Anxiety: + (Compared to both Placebo and Control)
(Cleland et al., 2006)	Asthma patients	92	46.26 (14.04)	62 (70.4)	Spiritual Healing	Placebo / Mock Healing Control	Week 4, 8, 12 & 24	HADS	Anxiety on week 12: + (Compared to both Placebo and Control)
(Cavalcante et al., 2016)	Anxiety	65	45.5 (15.5)	28 (76)	Spiritual Passe	Placebo / Mock Healing	Week 8	TAI, BDI	Depression: ND Anxiety: +
(Gerard et al., 2003)	Volunteers with restricted neck movement	68	53.2 (NA)	46 (67.65)	Prayer, meditation and the laying-on of hands	Control	Week 3	HADS	Depression: ND Anxiety: ND
(Jain, 2009)	Breast cancer	31	52.5 (NA)	31 (100)	Energy Healing	Placebo / Mock Healing	Week 4	CESD-R	Depression: +
(Miranda et al., 2020)	Breast cancer with depression and anxiety	31	50.95 (8.55)	31 (100)	Intercessory Prayer	Control	Week 4	HADS	Depression: ND Anxiety: ND
(Nikfarjam et al., 2018)	Anxiety	72	31.8 (10.22)	42 (58.34)	Teaching religious concepts repentance, prayers, recitation of Holy Quran and spiritual meditation (along with drug therapy)	Drug Therapy with no religious teaching	Week 3	STAI	Anxiety: +
(Palmer, 1998)	Individuals attending church	127	37.89 (9.84)	78 (61.52)	Lay Pastoral Telecare	Control	Week 24	CSA	Depression: +
(Rajagopal et al., 2018)	Mild to moderate depression	60	34.4 (NA)	31 (59.61)	Pranic Healing with Medicine	Placebo / Mock Pranic Healing with Medication	Week 4	HAMS-D	Depression: +
(Shore, 2002)	Depression	45	NA	NA	Hands On Reiki	Distance Non-Touch Reiki Placebo / Mock Reiki	Week 6	BDI	Depression: + (Compared to Placebo)
(Sundblom et al., 1994)	Idiopathic pain syndrome with depression	24	51.35 (NA)	12 (50)	Spiritual Healing	Control	Week 2	BDI	Depression: ND
(dos Santos et al., 2021)	Knee osteoarthritis	120	69.26 (5.29)	120 (100)	Spiritual Passe	Placebo / Mock Healing Control	Week 8	HADS	Depression: + Anxiety: + (Compared to Control Group)

*Note.* BDS = Beck Depression Scale; CESD-R = Centre for Epidemiological Studies Depression Scale-revised; CMD = Common Mental Disorders; CSA = Conversional Symptoms Assessment; DASS = Depression Anxiety Stress Scale; HADS = Hospital Anxiety Depression Scale; HAMS-D = Hamilton Depression Scale; HAMS-A = Hamilton Anxiety Scale; M = Mean; N = Numbers of participants; NA = Not available or not reported in source; ND = No Difference; SD = Standard Deviation; STAI = State Trait Anxiety Inventory; TAI = Trait Anxiety Inventory; + = Improvement of symptoms in intervention group.

#### Results of individual studies focusing on depression

Fifteen studies measured depression. Ten of these studies found that symptoms of depression improved among individuals attending interventions provided by traditional healers when compared to control or placebo groups; four studies indicated no difference. Cleland and colleagues (2006) neither provided descriptive statistics nor results in narratives. Miranda and colleagues (2020) did not provide statistics but reported that no significant differences were observed in depression between individuals attending traditional healers and the control group on the post-test. Two studies provided the difference in mean change scores (from baseline to outcome); Abbot and colleagues (2001) reported non-significant findings with no difference between the intervention and control group; however, dos Santos and colleagues (2021) reported significant improvement in the intervention group compared to the control group by week eight.

#### Quantitative synthesis on depression

**Figure 1.**
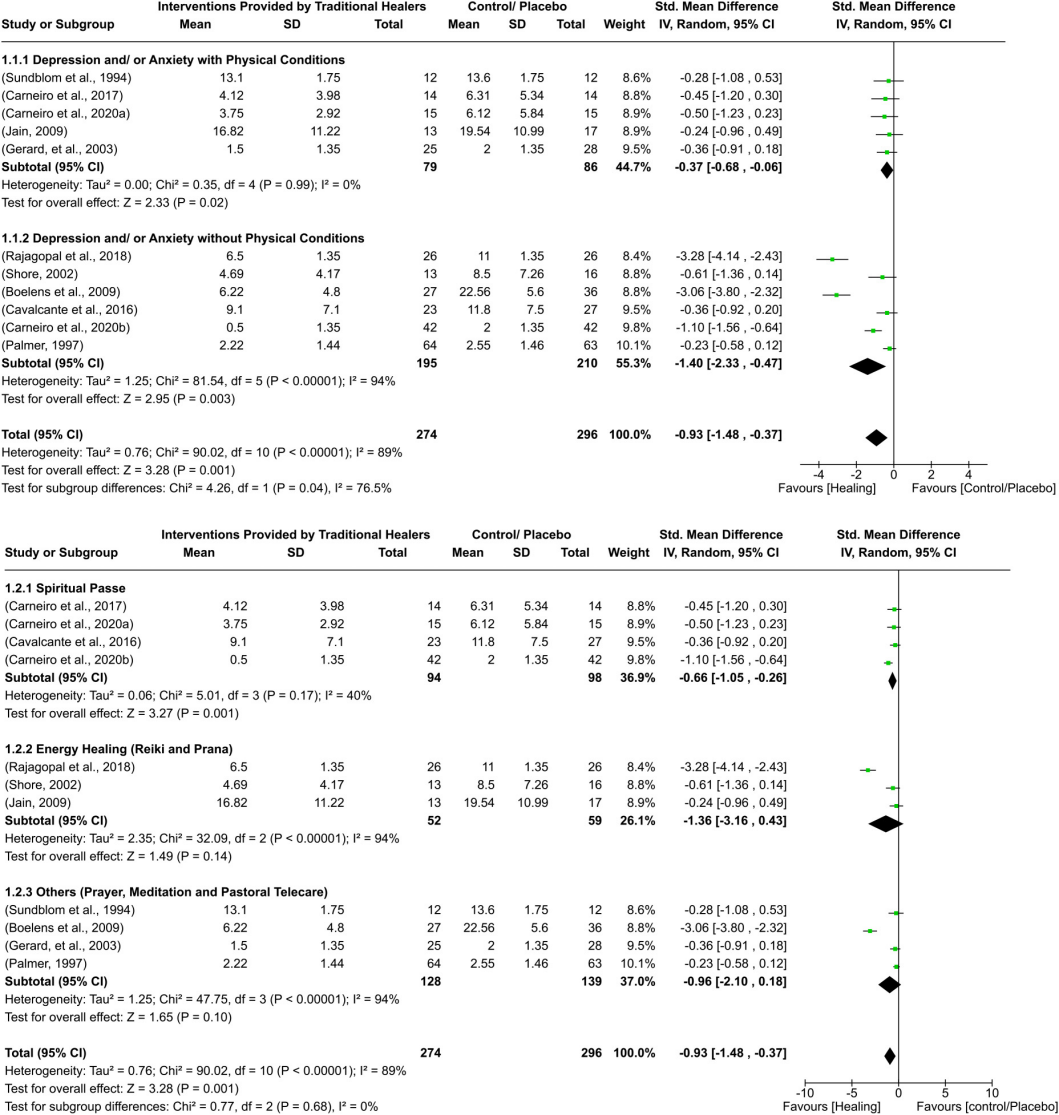
Effectiveness of traditional healing compared to control or placebo for depression on all-time outcome (with subgroups comparison based on co-existing physical conditions and intervention types).

**Figure 2.**
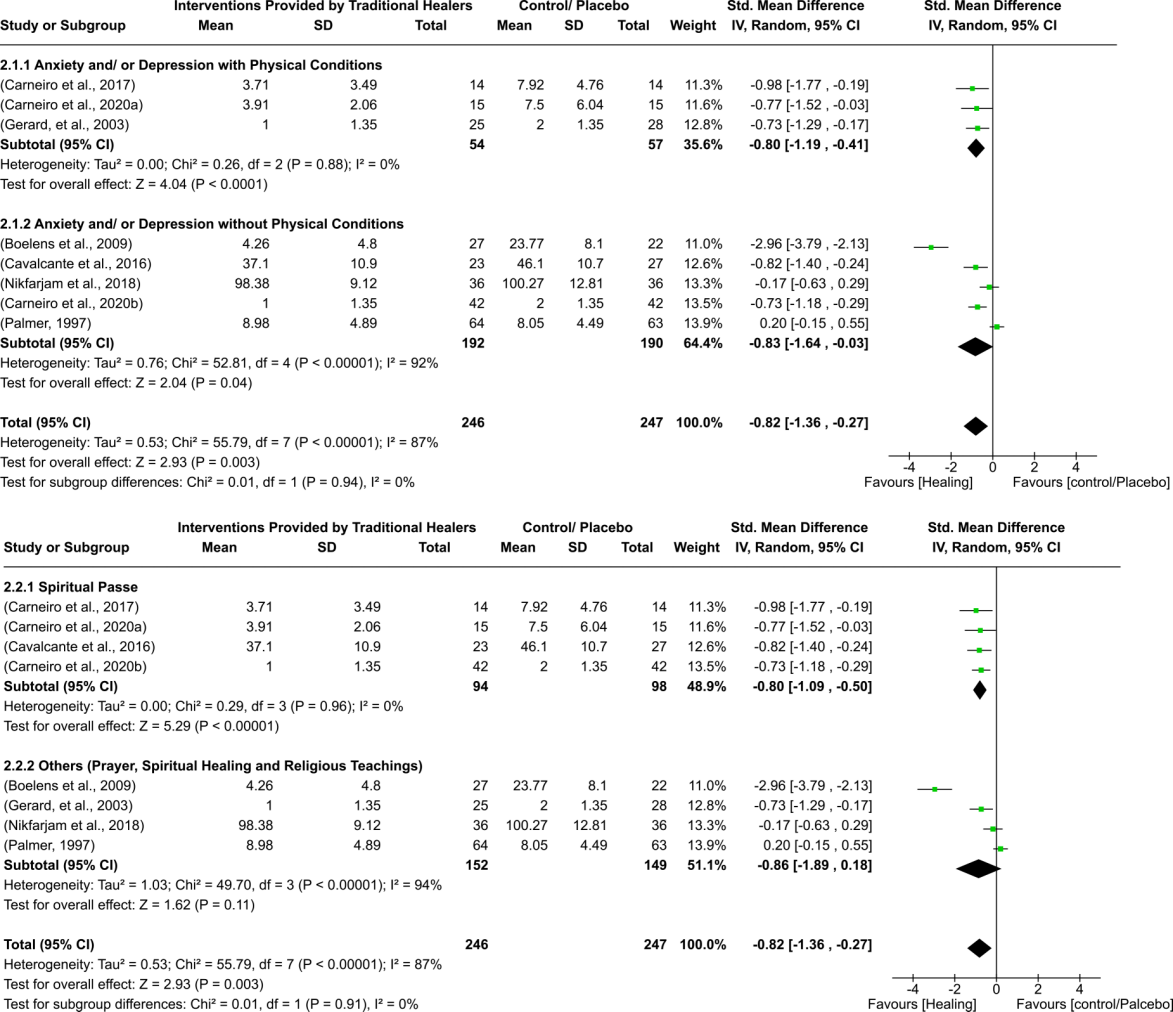
Effectiveness of traditional healing compared to control or placebo for anxiety on all-time outcome (with subgroups comparison based on co-existing physical conditions and intervention types).

The overall quantitative synthesis of 11 studies included 570 participants, with 274 randomised to interventions provided by traditional healers and 296 randomised to a control or placebo group. Findings demonstrated significant improvement in depression symptoms for individuals in the intervention group (standardised mean difference = −0.93; 95% confidence interval = −1.48 to −0.37) compared to control/placebo groups. A significant amount of variability was found (I^2 ^= 89%) (Figures 1 and 2).

#### Subgroup analysis based on co-existing physical conditions

Five of the studies looked at depression in people who had co-morbid physical conditions, and six studies on depression did not identify people with co-morbid physical conditions. Five studies on depression with physical conditions, spiritual passe, or a form of energy or spiritual healing were used. However, the studies on depression without physical conditions included a range of interventions including spiritual passe, energy healing, informal counselling, prayer, repentance, and meditation. Individuals randomised to the intervention group had significantly lower depression symptoms compared to control for both subgroups. However, the difference was greater for the depression without any physical conditions subgroup, and heterogeneity in the estimates of the studies within the depression with physical conditions subgroup was less (Figure 1).

#### Subgroup analysis based on types of intervention

Four studies were included in the spiritual passe subgroup, three in energy healing, and four in other interventions (such as prayer, meditation etc.). Intervention groups were significantly lower on depression symptoms compared to control only for the spiritual passe intervention. No statistically significant differences were observed between interventions and control for energy healing and other types of healing (Figure 1).

#### Sensitivity analysis

Three studies (Carneiro, Tosta et al., 2020; Gerard et al., 2003; Rajagopal et al., 2018) providing only medians and interquartile ranges were included in the meta-analysis. To rule out any possibility of bias, these three studies were removed. The overall effect favouring interventions by traditional healers compared to control remained significant. However, no significant improvements in individuals attending traditional healers for subgroups including depression with physical conditions and depression without physical conditions were observed. For subgroups based on intervention type, there was no effect of removing these three trials; the difference favouring interventions compared to control for the spiritual passe subgroup remained significant, while for other subgroups no difference was observed.

#### Results of individual studies focusing on anxiety

Anxiety was measured either as a primary or secondary outcome in 12 studies. Six studies reported low levels of anxiety symptoms among individuals attending traditional healers compared to control, whilst five studies reported non-significant findings. Two studies (Jain, 2009; Miranda et al., 2020) did not provide any descriptive statistics. Cleland and colleagues (2006) reported fewer participants positive on anxiety in week 12 in the intervention group compared to control, whilst Miranda and colleagues (2020) reported non-significant differences. Two studies found changes in mean scores; Abbot and colleagues (2001) reported non-significant findings, whilst dos Santos and colleagues (2021) reported significantly lower levels of anxiety among intervention compared to control in week eight.

#### Quantitative synthesis on anxiety

The quantitative synthesis of eight studies included 493 participants, with 246 randomised to interventions and 247 randomised to control/placebo groups. Findings demonstrated an improvement in anxiety symptoms among individuals attending traditional healers (standardised mean difference = −0.82; 95% confidence interval = −1.36 to −0.27) compared to control/placebo groups. A significant amount of variability was found (I^2 ^= 87%) (Figures 1 and 2).

#### Subgroup analysis based on co-existing physical conditions

Three studies were included in the anxiety with physical conditions subgroup and five were included in the anxiety without physical conditions subgroup. All studies in the anxiety with physical conditions subgroup used spiritual passe intervention; interventions in the anxiety without physical conditions subgroup were heterogenous, including spiritual passe, prayer, and meditation. This subgroup analysis indicated significantly lower scores in anxiety among individuals who received interventions compared to control for both subgroups. The estimates of the studies within the anxiety with physical conditions subgroup were more homogenous as compared to the estimates of the studies included in the without physical conditions subgroup (Figure 2).

#### Subgroup analysis based on types of interventions

There were three studies in the spiritual passe subgroup and four studies in the other interventions subgroup. There were lower levels of anxiety symptoms among the intervention compared to the control group in the spiritual passe subgroup. No significant differences were observed for other interventions subgroup. The estimates of studies within the spiritual passe subgroup were more homogenous compared to the other interventions subgroup (such as prayers and meditation etc.) (Figure 2).

#### Sensitivity analysis

Two studies providing median and interquartile ranges were included in the meta-analysis for anxiety outcomes (Carneiro, Oliveira et al., 2020; Gerard et al., 2003). By excluding the two studies, the difference between intervention and control remained significant for the anxiety with physical conditions subgroup, however the difference in favour of intervention vs control for the anxiety without physical conditions subgroup was no longer significant. Removal of the two studies did not have any effect on estimates for the spiritual passe and other interventions subgroup.

#### Risk of bias

The overall quality of the studies was generally low, with 13 studies rated either as “low” or “very low.” Three studies were rated as “moderate” quality by the reviewers (Table 1). Seven studies (43%) were at high risk of bias either due to incomplete data or attrition bias, and five studies (30%) were at high risk for performance bias. Fourteen studies (90%) were unclear about allocation concealment, 12 studies (75%) were unclear about the blinding of participants and personals and the blinding of outcome assessors, and nine studies were unclear about selective reporting (about 55%) (Appendix D). The figure presented in Appendix E indicates potential publication bias with an uneven distribution of studies.

### Barriers and facilitators

#### Study characteristics

The synthesis incorporated qualitative findings from 12 qualitative studies and five mixed-methods studies. Twelve studies were conducted in non-healthcare settings (including in the community, mosques, and faith-based institutions) and four studies were conducted in healthcare settings. Eleven studies focused on the views and perspectives of traditional healers towards CMDs and six studies explored the experiences of participants with CMDs who received interventions from traditional healers (Tables 1 and 3). The studies were based in the following countries: 11 studies were based in the United States and the remaining six studies were conducted in Ghana, Brazil, South Africa, Liberia, Canada, and Somalia.

**Table 3.** Participants’ characteristics, focus, key findings, and quality of the studies included in synthesis.

Study ID	Characteristics of participants	Number of participants	Females N (%)	Age range / Age M (SD)	Topic of interest
(Stansbury et al., 2009)	Baptist Clergy/ Pastors	9	0 (0%)	36 to 58	Views on experience in dealing with depression in elder congregants
(Bryant et al., 2014)	Pastors	9	0 (0%)	Not Given	Knowledge, attitude, and perspective about depression
(Hankerson et al., 2021)	Clergy Members	2	1 (50%)	27 to 42	Perspectives on training clergy members in an evidence-based intervention for depression
(Jang et al., 2017)	Clergy Members	17	1 (5.9%)	35 to 87/ 52.4 (14.3)	Awareness and literacy related to depression
(Andren & McKibbin, 2018)	Clergy Members	126	Not Given	19 to 80/ 58.24 (9.28)	Referral choice for depression
(Kpobi & Swartz, 2018)	Traditional and Faith Healers	36	Not Given	Not Given	Views and perspectives on depression
(Lucchetti et al., 2015)	Patients with depression	2	1 (50%)	26 to 34	Questions related to experience of treatment provide by traditional healers
(Moodley et al., 2018)	Muslim Faith Healers / Islamic Scholars	5	0 (0%)	30 to 69	Understanding of mental illness, depression, treatment, and collaboration with medical professionals
(Payne & Hays, 2016)	Christian Clergy and Pastors	35	Not Given	Not Given	Management strategies for individuals with depression, suicidal ideation, PTSD, and anxiety
(Pullen et al., 2021)	Traditional healers and utilisers of traditional medicine	35	9 (37.5)	Not Given	Common healthcare and psychosocial problems, attitude towards western medicine, treatment provided by traditional healers
(Pyne et al., 2021)	Veterans with Post-Traumatic Stress Disorder; Community Clergy Members	13	4 (31%)	56.8 (8.8)	Barrier and facilitators related to PTSD intervention facilitated by clergy members
(Pyne et al., 2019)	Veterans with Post-Traumatic Stress Disorder; Community Clergy Members and Mental Health Clinicians	39	6 (5.4)	49.06 (10.9)	Veteran experience talking with clergy, and clergy clinician experience talking with veterans about spiritual issues and related barriers and facilitators
(Stansbury, 2011)	Baptist Pastors	9	0 (0%)	47 to 68	Awareness, causes, and treatment for depression
(Storck et al., 2000)	Depression	3	2 (66.6)	47 to 64	Understanding of illness, traditional and religious interventions
(Tobah, 2017)	Muslim Leaders (Who lead prayer in Mosque)	8	Not Given	Not Given	Difference between professional mental health organisations’ illustrations and opinions of religious leaders to mental health, mental illness, and depression
(Wahbeh et al., 2017)	Post-Traumatic Stress Disorder	6	0 (0%)	27 to 67/ 49.3 (13.1)	Views regarding intervention related to PTSD
(Zoellner et al., 2021)	Post-Traumatic Stress Disorder and Muslim Leaders	26	14 (53.9)	18 to 47/ 27.64 (7.35)	Views regarding Islamic Trauma Healing in intervention, barriers, and facilitators

#### Traditional healers’ identified facilitators

*Religious and faith-based worldview*: The overall attitude of both Christian and Muslim traditional healers was inclined towards a religious explanation of mental illness. The explanation of why the mental illness happens was reported with reference to religious scriptures such as the Bible and the Quran. The focus was on the spiritual nature of humans, emphasising the importance of the spiritual self and God in the healing of mental illness. While the care provided by traditional healers included talking with individuals and listening to their concerns, the discussion between traditional healers and individuals is grounded in religious, spiritual, and faith-based worldviews.

I provide care from a spiritual perspective, meeting with them, hearing their concerns, letting talk through that, offering alternatives that may be more of a spiritual nature or biblical nature than what a secular counsellor might offer. (Stansbury et al., 2009, p. 12)

It was explicitly pointed out that such approaches are distinct from the secular counselling provided by professional healthcare providers. In one instance, traditional healers indicated that religious people are less prone to mental illness, without differentiating between the religions.

I don’t know much about depression, but I do know that religious people do not get depressed. You know that a famous Korean actress committed suicide a few years ago because of her depression. I heard she was Christian. But I don’t believe so. People deeply rooted in religion would never get depressed. (Jang et al., 2017, p. 389)

For traditional healers and their attendees, it was seen as imperative to incorporate religious values and beliefs into mental health interventions, as it is critical to their belief system. The choice and preference for choosing traditional healers for religious communities are shaped by their faith and religious values.

*Knowledge of mental illness and risk factors*: Some traditional healers were aware of the signs and symptoms of depression, and used terminologies such as “hopelessness,” “disinterest,” “sadness,” and “detachment” to portray depression.

I feel like for me there's indicators of bad state and what I tend to see is that they, they don’t have much hope. They’re not … they’ve become disinterested in certain things in life. They … you know, the way that they’re talking is almost monotone … you know, detached. It's not so much about solutions anymore. Those are things that I find very concerning. (Tobah, 2017, p. 53)

A psychosocial explanation was apparent in the data, and traditional healers reported that lack of interest and detachment from life activities as the significant indicators of depression. Risk factors for depression such as the loss of a spouse were also identified by traditional healers: “I worry about my elders who lose their spouse of forty, ﬁfty, or sometimes sixty years. In my line of work, I ﬁnd that when an older person loses their spouse, they become depressed and despondent” (Stansbury, 2011, p. 303). The difference between depression and severe mental illness (referred to as madness) was acknowledged by traditional healers.

Yes, that is also a mental problem, but it is not like the madness … I think you people call it depression … it often happens with ladies. I’m sure the lady has many problems in her life, and she can’t cope. So, she will be thinking about it all the time … and then it makes her sad. (Kpobi & Swartz, 2018, p. 6)

While it was apparent that traditional healers were aware of indicators and associated factors of depression, it is also apparent that this is due to exposure rather than formalised training. As indicated above, the language of depression is somewhat different to a more medicalised language, and differentiations between different forms of CMD were not acknowledged.

*Distrust in statutory mental health approaches*: Distrust in formal services was considered a facilitator to CMD care being accessed through traditional healers, as it meant people with CMD were more likely to see the religious healers and trust them for advice than formal services. A distrust in the effectiveness of professional mental health services has been apparent in traditional healers’ responses in two studies (Stansbury et al., 2009): “It may not be all time that they may need that professional help because sometimes the professional help doesn’t help sometimes so most of the time, I try not to send them to professional help” (Stansbury et al., 2009, p. 12). Both studies involved Baptist pastors in the United States. They explicitly reported that they do not prefer to advise individuals to consult healthcare professionals. The explanation provided for not advising individuals to consult healthcare providers was that medical practices rely on prescribing medicine for mental illness which is often ineffective.

There are some good psychiatrists, therapists, social workers, doctors. I just do not know of them. It seems to me that all these mental health practitioners want to do is give pills to everyone with mental health problems. Just take a pill and everything will be okay. Sometimes good old talk therapy is all an elder needs to snap back into reality. I do not believe in medication because the medicine turns them into zombies to a point where they do not know if they are coming or going. (Stansbury, 2011, p. 305)

Distrust may relate to not having a medical explanation of illness. Additionally, a potential lack of contact between traditional healers and health professionals, and perceptions of professional healthcare providers over-prescribing for mental problems were reported by traditional healers. In both studies, the traditional healers did not report any psychosocial strategies utilised by healthcare providers.

*Use of informal counselling and guidance*: As ‘sadness’ or depression was recognised, so informal care provided through talking was reported by healers. Informal psychosocial care such as the use of guidance through talking and teaching activation strategies was used by traditional healers, e.g., advice to become involved in activities.

I got her to take part in activities and that is one thing to get her not to look down or become depressed and not have the will to live. I think I always encouraged her to be more active, but don’t just stay home because that brings on more depression but getting out and going and getting members to encourage her also. (Stansbury et al., 2009, p. 12)

The psychosocial explanation for depression was also apparent and it was explicitly mentioned that “staying at home” exacerbates the symptoms of depression. Becoming active in daily life activities, especially involvement in religious activities such as attending prayers at church and mosques, was encouraged by religions in the study. Such explanations as to how to alleviate one's mood are common grounds between healers and health professionals.

*Community engagement in religious institutions*: Involvement of religious institutions with community members was encouraged and members were invited to a range of church activities – such as prayers, community gatherings, and volunteer support groups for immigrants. This indicates that a proactive approach has been utilised to approach individuals by traditional healers: “Mainly through personal home visits, encouraging church members to do likewise, always including her and keeping her informed about church activities” (Stansbury et al., 2009, p. 12). It should be noted that most traditional healers in the study are not only healers but also religious and community leaders who have a wider role in society. People approach them not only for their mental illness but also for their other personal problems and for religious and spiritual guidance (even if they have no mental illness). For instance, one traditional healer discussed providing practical support to immigrants:

We are not only clergy members but also social service providers. From their arrival to settlement, we provide all kinds of help and information to newcomers. When immigrant families arrive, we are there at the airport and help them settle. (Jang et al., 2017, p. 391)

A proactive and community-based approach utilised by traditional healers has increased the accessibility and availability of such services to the community. Making connections by visiting home and helping people is probably one the most important elements which shape the preference of individuals with mental illness to visit traditional healers.

#### Traditional healers’ identified barriers

*Stigma and silence regarding mental illness*: Stigma associated with mental illnesses for minorities (including Korean immigrants settled in the United States and Black community members in the United States) was reported by traditional healers in two studies (Bryant et al., 2014; Jang et al., 2017). For instance, it was highlighted that among a Korean community in the United States, admitting having mental health issues is often seen as shameful by family and community members: “Mental health problems are perceived as a shame and something that should not be disclosed to other than family members” (Jang et al., 2017, p. 390). In another study, a traditional healer explained that in the Black community in the United States, depression is often considered a sign of weakness, which may make people reluctant to seek help from professional healthcare workers and to visit traditional healers.

Admitting depression for a man, especially a Black man, is admitting weakness … I’m going to ﬁnd a way gets it [depression] ﬁxed. That's admitting I’m weak, and I can control it [depression]. As black men, we don’t do it [admit depression]. (Bryant et al., 2014, p. 803)

In both these studies, rather than sharing or discussing mental illness, individuals with mental illness tend to try to manage it by themselves, which includes not seeking help from traditional healers. Furthermore, in these studies, it was reported to be common practice to hide emotions in general and to teach children not to cry when upset. Family values and norms also play a significant role in recognising and pursuing help for mental illness. Again, this reflects that such family beliefs create hindrances in accessing professional and informal help (such as traditional healers). This especially applies to vulnerable communities with collectivistic family norms.

*Lack of training and capacity building*: Despite distrust towards formal services in two of the studies and some knowledge of depression in most of the studies, healers explicitly stated that they needed extra training as they were not confident in detecting cases of depression. Any knowledge of depression was a result of their personal exposure and experience with cases of depression, rather than a confidence in being able to categorically identify depression: “I don’t think there is one easy and simple way to say what depression is. I am personally not quite sure if I can correctly detect someone who has depression” (Jang et al., 2017, p. 388). Furthermore, most traditional healers reported that they do not feel prepared to properly manage people's mental illness, and in some studies the presence of professional counsellors in the church was identified as a helpful way to build capacity (Hankerson et al., 2021; Jang et al., 2017): “I [clergy member] don’t necessarily feel the most prepared to do [depression counselling]. I do it, but yeah, I don’t necessarily feel as prepared as I should be” (Hankerson et al., 2021, p. 975).

*Openness towards medical and statutory mental health care*: It has been observed across multiple studies that traditional healers were open towards statutory mental health services for their followers and congregants, except in two studies discussed above under distrust in the medical approach. In these studies, traditional healers acknowledge the role of medical factors such as chemical imbalance in mental illness and accordingly believe that it cannot be fixed through healing strategies such as prayer. It was apparent in the data that healers reporting openness towards a medical approach were aware of basic medical terminologies, indicating an exposure to health professionals: “How would you ﬁx a hormonal problem with prayer? It is impossible. You need to see a mental health specialist and get medical treatment” (Jang et al., 2017, p. 390).

The limitations on practices of traditional healers were also acknowledged by healers, where they explicitly pointed out that medical problems are not their area of expertise and they don’t have sufficient training for them: “I feel that I am equipped to address spiritual needs; however, if there is a need for medical treatment or a medication, those are areas in which I have not been trained” (Andren & McKibbin, 2018, p. 111). Additionally, in one of the studies, Muslim traditional healers reported advising people to seek medical treatment when necessary: “We advise them to go for medical treatment to psychiatrist or psychologist because we are not qualified in that area” (Moodley et al., 2018, p. 89). This theme is identified as a barrier, as it may mean individuals are less likely to visit traditional healers. However, the studies examining where healers are open to medical healthcare indicate that individuals may use both services and that there is scope for collaboration.

#### Traditional healers’ attendees’ identified facilitators

*Inclination towards faith and religion*: Faith-related activities and a focus on spirituality were apparent in the responses of the people with CMD attending traditional healers. Traditional healers’ attendees reported leaving problems to God and that faith helps in alleviating mental illness. They reported that they have to rely on religious rituals such as prayers with pure intention and devotion. For building faith in God, prayer was identified as a beneficial activity: “When I got saved, I had to say my own prayer from my heart, from my inner self to just give everything, all my problems back to the Lord and let Him take care of it” (Storck et al., 2000, p. 584). We noted that historical accounts preserved in religious texts serve as an enduring source of inspiration for people attending traditional healers. These narratives provided a sense of relation, where people can draw parallels between their own difficulties and the adversities overcome by their elders. As pointed out by traditional healers, their practices are guided by such texts such as the Quran and the Bible. The holy books contain accounts of Prophets (messengers of God), such as their difficulties and ways in which they coped with these difficulties. Such coping behaviours utilised by Prophets were considered as guidance for followers—“What I most liked about this program was the stories of the Prophets”—and appreciation was expressed for “making healing from the Islamic religion” (Zoellner et al., 2021, p. 5). This theme converges with the theme and responses reported by traditional healers, and it indicates that traditional healers and individuals who attend those healers have shared values and beliefs which shape the preferences of individuals seeking help for mental illnesses (Zoellner et al., 2021).

*Helpfulness of religious interventions*: We noted that attending religious ceremonies, prayers, and other faith-related activities offers an opportunity for emotional catharsis and healing through communal gathering and shared spirituality. Experiences while attending such ceremonies and going through the healing process reflect the transformative process as well as adverse experiences felt during mental illness. People attending traditional healers reported that healing remedies help in improving well-being and relationships with family members (Storck et al., 2000).

Attended the meeting and cried my heart out for my late father. I felt like something was crushing my chest and that it was wrapped all around my chest as hard as you could tighten it, but at this meeting the tightness softened up and then I settled down and that is how I am today. (Storck et al., 2000, p. 580)

They tend to relate their experience of mental illnesses and their transformation phase to the heart rather than the mind. Among individuals who saw traditional healers, there was a reference to one's “heart” or “chest” which would relax due to religious interventions: “My heart continues to soften. It is much easier to see people as individuals. While I have ﬂeeting moments and ﬂashes on negative circumstances, I care and feel better about myself” (Wahbeh et al., 2017, p. 212). In many religious scriptures, “heart” is considered a controlling unit of the body, thus traditional healers using culturally relevant terminologies allows individuals with mental illness to express their beliefs in a relevant and meaningful way.

#### Traditional healers’ attendees’ identified barriers

*Contextual and logistic barriers*: We observed that accessibility to traditional healers remained a challenge due to constraints on their time and resource limitations. Contextual and logistic barriers to accessing traditional healers included the healers not having enough time to spend with them: “Three months just wasn’t enough for me” (Pyne et al., 2021, p. 3042). In one of the studies, an individual attending a traditional healer exhibited concern about confidentiality and privacy, especially in informal settings, where traditional healers lack a proper space to ensure privacy and confidentiality (Pyne et al., 2021): “I do not want my business on the street” (Pyne et al., 2019, p. 9).

*Training and awareness barriers*: As with traditional healers, their attendees also identified a lack of formal training in mental health for traditional healers: “Not be too quick to think you understand. You have not been there. Every veteran's story is different” (Pyne et al., 2019, p. 10). They reported that traditional healers need to understand the problems in greater depth and address them on a case-by-case basis.

#### High-income vs. low-income regions

Most of the studies included in the qualitative synthesis were based in HICs (N = 11), with only three based in low-resource settings. Most barriers and facilitators identified were limited to HIC. Traditional healers’ identified facilitators, including knowledge of mental illnesses and use of informal guidance, were observed in studies based in LMICs including Ghana and Liberia (Kpobi & Swartz, 2018; Pullen et al., 2021), as well as in HICs. Further, attendees’ identified facilitators were observed in both HICs and LMICs.

#### Risk of bias

The quality of the qualitative or mixed-methods studies was mostly moderate to low; seven studies were rated as moderate quality, seven as poor quality, and three as good quality (see Table 1). Generally, the studies used an appropriate method for data collection and provided a clear rationale for their methodology and recruitment. However, there was little evidence of reflexivity being addressed in most of the studies. Additionally, several studies did not provide comprehensive details about data analysis.

## Discussion

This is the first review to date which is based on a synthesis of RCTs together with a review of barriers and facilitators regarding the application of interventions delivered by traditional healers for CMD. Overall, we found that high-quality evidence was limited. Most of the studies on the effectiveness of interventions by traditional healers were carried out in HICs. Given the considerable proportion of people attending traditional healing for mental health (Burns & Tomita, 2015; Khan et al., 2019), coupled with the lack of trained human resources and facilities for mental health care in these countries (World Health Organization, 2009), it is worth considering why this area has been overlooked in LMICs (Rathod et al., 2017). This is particularly important, considering the large mental health treatment gap, and that previous evidence suggests interventions offered by traditional healers may be effective for treating depression and anxiety (Nortje et al., 2016). There is also some evidence demonstrating the willingness of both traditional healers and healthcare professionals to collaborate (Green & Colucci, 2020).

Overall, the present review suggests that interventions provided by traditional healers improved the symptoms of depression and anxiety compared to control groups. However, subgroup analysis together with sensitivity analysis further suggested that interventions provided by traditional healers improved symptoms of anxiety only in individuals with co-existing physical conditions. Further, spiritual passe interventions also improved symptoms of depression and anxiety, whereas other interventions (meditation, prayer etc.) demonstrated non-conclusive findings. The effectiveness of spiritual passe interventions with more precise estimates for each study reflects that it is a well-explored intervention in medical care with consistent methodological approaches, geographical location, and homogeneous participant characteristics. Intervention characteristics of spiritual passe may potentially be another characteristic where procedures and ways of performing spiritual passe are well defined. However, it should be noted that outcomes of anxiety in the co-existing physical conditions and spiritual passe subgroups were mostly measured up to only three days after delivering interventions, therefore findings about improvements in depression and anxiety for spiritual passe interventions and improvements in anxiety for participants with physical conditions are largely limited by the premature timing of the outcome measurement.

Studies evaluating other interventions (prayer, repentance, religious teaching etc.) were inconclusive, largely heterogenous, and fewer (five studies) in this review. Interventions such as prayers, repentance, meditation, and religious teachings may vary due to different religious and cultural values, therefore inconclusive findings identified by this review may warrant more clarity around defining different traditional healing approaches other than spiritual passe. Additionally, compared to the outcome timings for the spiritual passe intervention, studies evaluating other interventions measured outcomes at considerably different timings, ranging from 4 to 12 weeks. Further, the studies in the effectiveness review were at higher risk for attrition bias, which concurs with the previous systematic review (Nortje et al., 2016).

There were differences in interventions that addressed CMD with co-existing physical conditions and CMD without physical conditions. All the studies including participants with CMD with co-existing physical conditions used either spiritual passe or another energy healing intervention, except for one study which utilised prayer. In comparison, a range of interventions were used for participants with CMD without physical conditions, including spiritual passe, energy healing, and worship traditions (such as prayers, repentance, and religious teaching). Spiritual passe and other forms of healing such as reiki or prana are based on the belief that a universal energy flows through the human body and the goal of healing is to bring balance to the energy flow in an individual body (Schmidt, 2021). Energy healing interventions have different names as they are different in different parts of the world and depend on different spiritual practices and traditions. For example, spiritual passe is widely practised in Western countries (Schmidt, 2021), whereas prana healing originated and is practised in India (Sui, 2015). Such interventions may be referred to as spiritual, however these energy healings are not well supported by religious traditions in religions such as Christianity as positioned by the Committee of Catholic Bishops (Bishops, 2009).

While there are differences in traditions of performing prayer, repentance, and meditation between religions, the most practised Abrahamic religions (Islam, Christianity, and Judaism) generally consider prayers, repentance, and meditation as integral parts of their worship traditions. For instance, Quranic and Biblical interpretations consider the soul and closeness to God as an integral part of human psychology and believe the soul can be purified through following the traditions of worship, thereby through the purification of the soul human beings become more connected to God and hence are less prone to psychological or mental illness (Jeppsen et al., 2022; Rothman & Coyle, 2018).

Our review has strengthened the existing evidence provided by previous reviews, further supporting the potential effectiveness of interventions delivered by traditional healers for anxiety (Nortje et al., 2016; Van der Watt et al., 2018). However, findings were also different in the case of depression, where we found no substantiated evidence for their effectiveness. An additional unique feature of our review was evidence indicating the beneficial effects of interventions by traditional healers on anxiety, even when it is accompanied by co-existing physical conditions. This highlights the potential versatility and broader applicability of such interventions in addressing mental health concerns within the context of comorbid physical health issues. However, it might not be true for all kinds of traditional healing and warrants further investigation considering heterogeneity related to interventions and long-term outcomes. Furthermore, qualitative and mixed-methods studies incorporated in the review provide further insight into the findings.

The findings from the qualitative synthesis were in line with the quantitative findings. People who had engaged in traditional healing reported that they felt better after attending religious ceremonies and traditional healing sessions. It was also apparent in the data from traditional healers that faith-based activities including believing in God, having a relationship with God, and attending church activities may act as a protective factor against common mental health issues such as depression. Data from intervention-based (Gonçalves et al., 2015) and correlational studies (Anderson & Nunnelley, 2016) have suggested similar findings whereby integrated faith-based approaches have been found to be more effective compared to non-faith-based psychosocial interventions (Anderson et al., 2015). Community engagement of traditional healers and religious institutions was well documented in the studies as traditional healers engaged in community-based activities. This is especially important in the context of facilitating and increasing access to treatment care. Stigma, especially related to minority groups, is a noteworthy barrier which may result in the suppression of mental health issues and of access to both formal and informal care.

Our findings were to some extent similar to previous reviews which provided some useful insights into factors related to collaboration between traditional healers and healthcare professionals (Green & Colucci, 2020). Some facilitators we identified were consistent with these previous reviews, such as a shared understanding of mental illnesses grounded in faith, enabling people to access traditional healers. However, our findings and samples were also different in certain aspects, as our review included perspectives from both traditional healers and their attendees. We identified relationship dynamics between traditional healers and their attendees, for example concerns related to privacy issues reported by attendees, whereas a previous review only focused on collaborative aspects, reporting the institutional dynamics and professional reputation, with a focus on the perspectives of traditional healers and healthcare providers (Green & Colucci, 2020). Primary data on the perspectives of people attending traditional healers are limited. In future studies, it would be important to address barriers to collaboration between traditional healers and healthcare providers based on the perspectives of people attending traditional healers. Although traditional healers were open to learning and undergoing training in formal mental health services such as psychosocial training, they showed distrust of medicine-related treatments. This perspective was also identified in previous reviews which reported that traditional healers often feel that their practices are seen as inferior by health professionals (Green & Colucci, 2020). Their openness towards psychosocial strategies was not only identified in their perspective but they also reported that they informally used psychosocial guidance through the use of activation strategies (by encouraging individuals to engage in adaptive behaviours). It was also found that informal care providers with a religious background which are often referred to as “traditional healers” (as in this review) or “faith healers” in the medical literature have a wider role in society. Developing a broader understanding of and wider terminologies for traditional healers could potentially facilitate mental health professionals to acknowledge (and tap into) the wider social network, following, and approachability of such healers.

## Strengths and limitations

We conducted a systematic review and meta-analysis, along with a narrative synthesis which comprehensively reviewed the evidence on the treatment of CMD by traditional healers and included perspectives of traditional healers and those attending them. We followed established and recommended methods for such evidence syntheses. Some limitations of the evidence, which limit definitive conclusions, such as the timing of outcome measurements, have been described above. In addition, participants in the included studies did not receive diagnoses of depressive or anxiety disorders. Rather, the presence of symptoms related to depression and anxiety was assessed using standardised measurement tools. As a result, the findings from our review may not be directly applicable to individuals who have been formally diagnosed with these disorders.

## Conclusion and implications

Our review provides evidence that interventions delivered by traditional healers improve symptoms of anxiety in individuals with co-existing physical conditions. Also, spiritual passe interventions improve symptoms of both depression and anxiety. However, these findings are largely limited by premature timings of outcome measurement in these studies. The qualitative data indicated that traditional healers and their attendees generally found traditional healing helpful for mental health. While stigma, lack of training of traditional healers, and lack of infrastructure or logistics were identified as significant barriers to traditional healers’ interventions for CMDs, facilitators included a faith-based worldview, some knowledge of CMDs, and an acknowledgement of a need for further training by traditional healers. An understanding of the barriers and facilitators is important if traditional healers are to be engaged in future interventions regarding CMDs.

## Supplemental Material

sj-docx-1-tps-10.1177_13634615241273001 - Supplemental material for Effectiveness, barriers, and facilitators of interventions delivered by traditional healers for the treatment of common mental disorders: A systematic review Supplemental material, sj-docx-1-tps-10.1177_13634615241273001 for Effectiveness, barriers, and facilitators of interventions delivered by traditional healers for the treatment of common mental disorders: A systematic review by Mujeeb Masud Bhatti, Najma Siddiqi, Hannah Jennings, Saima Afaq, Aatik Arsh, and Bilal Ahmed Khan in Transcultural Psychiatry
