# Supplementary material for: Effectiveness, barriers, and facilitators of interventions delivered by traditional healers for the treatment of common mental disorders: A systematic review
Source: Transcult Psychiatry. 2024 Sep 24;61(6):885–904. doi: 10.1177/13634615241273001 (PMC11664887; doi:10.1177/13634615241273001)
Supplement: sj-docx-1-tps-10.1177_13634615241273001 - Supplemental material for Effectiveness, barriers, and facilitators of interventions delivered by traditional healers for the treatment of common mental disorders: A systematic review [file sj-docx-1-tps-10.1177_13634615241273001.docx]

# Supplementary Material

**Appendix A: Screening Form**

| **Inclusion Criteria** | |
| --- | --- |
| 1 | Participants who have experienced or concurrently experiencing any intervention from traditional healers or faith healers^[[1]](#footnote-1)^ (in collaboration with biomedical or trained by biomedical professionals or independently) for symptoms of Common Mental Disorders (CMD) [including Anxiety Disorder, Panic Disorder, Depression, Obsessive Compulsive Disorder (OCD) and Post Traumatic Stress Disorder (PTSD)] with or without any physical or other mental conditions |
| 2 | Traditional healers or faith healers talk about any intervention (in collaboration with biomedical or trained by bio-medical professionals or independently), they provide to people with CMD |
| 3 | The study is based: |
| a) | Either on Randomised Controlled Trial (RCT) design with or without qualitative elements |
| b) | Or any qualitative & quantitative study with qualitative elements/ quotes |
| 4 | Studies with qualitative elements reporting the experiences, perspectives or opinions of patients, traditional and faith healers relating to any intervention provided by traditional and faith healers |
| **Exclusion Criteria** | |
| 1 | Traditional or Faith healers prescribing some oral or topical or nasal or inhaling herbal/ chemical/ substances |
| 2 | Studies including multiple interventions from traditional/ faith healers, and it is not possible to differentiate psychosocial interventions (including prayer, recitation, etc.) from herbal/ chemical/ substance-based intervention |
| 3 | Studies in which either participants with both CMD and severe mental disorders or participants with dual diagnosis were included, and it is not possible to differentiate quantitative outcomes or qualitative responses (quotes) of participants with common mental disorders from participants with severe mental disorders |

**Appendix B: Search Strategy**

|  |  | **Search Terms** |
| --- | --- | --- |
|  | Participants | Sangoma OR Curander* OR Spiritis* OR Spiritualis* OR Voodoo OR Voudou OR Santeria OR Divination OR Diviner OR Shaman* OR Clergy OR Witchdoctor OR "Witch-Doctor" OR "Faith Heal*" OR Faith healing/ OR "Indigenous Heal*" OR "Traditional Heal*" OR "Native Heal*" OR "Religious Heal*" OR "Religious leader" OR "Ritual Heal*" OR "Spiritual Heal*" OR Marabout OR Dervish OR Sheikh OR Sufi OR Imam |
|  | Intervention | ------ |
|  | Comparison | ----- |
|  | Outcome | Mental disorder/ OR Mental OR Psychiatr* OR "Common Mental Disorders" OR Depress* OR Anxi* OR Panic OR "Obsessive Compulsive" OR "Obsessive-Compulsive" OR OCD OR "Post Traumatic Stress" OR "Post-Traumatic-Stress" Or PTSD |
|  | Study Method (Mixed Method Filter) | Case Reports/ OR Organizational Case Studies/ OR Qualitative Research/ OR qualitative research* OR qualitative stud* OR action research OR Community-Based Participatory Research/ OR participatory research OR case stud* OR ethno* OR grounded theory OR phenomeno* OR Narration/ OR narrative* OR biograph* OR Autobiography/ OR Autobiograph* OR documentar* OR qualitative synthes* OR active feedback OR conversation* OR discourse* OR thematic OR qualitative data OR key informant* OR Focus Groups/ OR focus group* OR case report* OR Interview/ OR interview* OR Observation/ OR observer* OR visual data OR (audio adj record*) OR Anthropology, Cultural/ OR experience* OR exp clinical trial/ OR exp Research Design/ OR random allocation/ OR double-blind method/ OR Single-Blind Method/ OR Placebos/ OR Cross-Over Studies/ OR (clinic* adj25 trial*) OR random* OR control* OR (latin adj square) OR placebo* OR Comparative Study/ OR comparative stud* OR Validation Studies/ OR validation stud* OR evaluation studies/ OR evaluation stud* OR Follow-Up Studies/ OR followup OR follow-up OR Prospective Studies/ OR Cross-Over Studies/ OR cross over OR crossover OR prospective* OR volunteer* OR ((singl* OR doubl* OR trebl* OR tripl*) AND (mask* OR blind*)) OR Cohort Studies/ OR Case-Control Studies/ OR Cross-Sectional Studies/ OR Health Surveys/ OR Health Care Surveys/ OR Risk/ OR Incidence/ OR Prevalence/ OR Mortality/ OR cohort* OR case-control OR cross sectional OR (health* adj2 survey*) OR risk OR incidence OR prevalence OR mortality.tw OR "case series" OR "time series" OR "before and after" OR prognos* OR predict* OR course* OR (mixed adj5 method*) OR multimethod* OR (multiple adj5 method*) OR ((qualitative) AND (Qualitative Research/ OR quantitative)) |

**Appendix C: PRISMA Flow Chart**


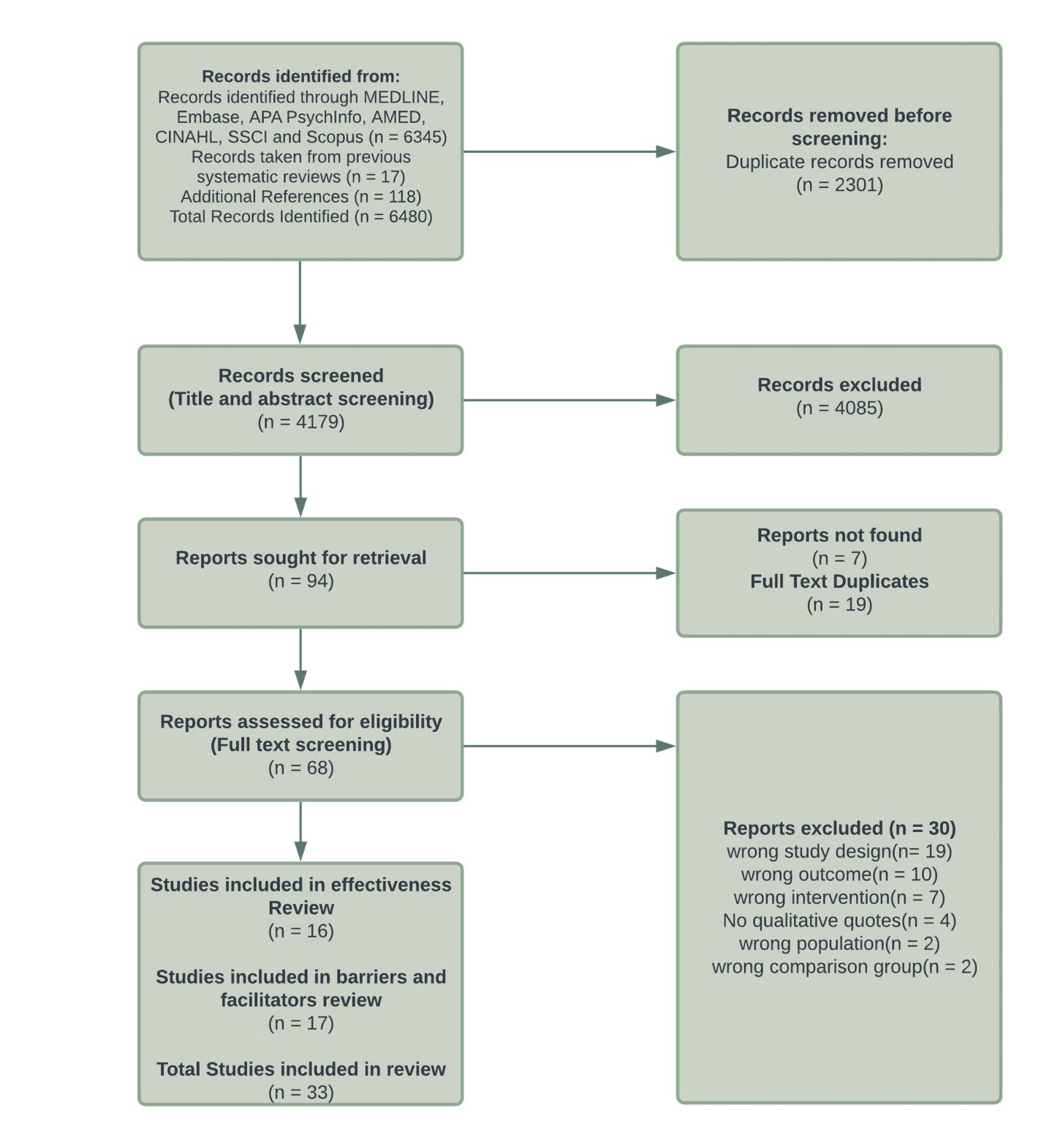


**Appendix D: Risk of Bias Assessment**


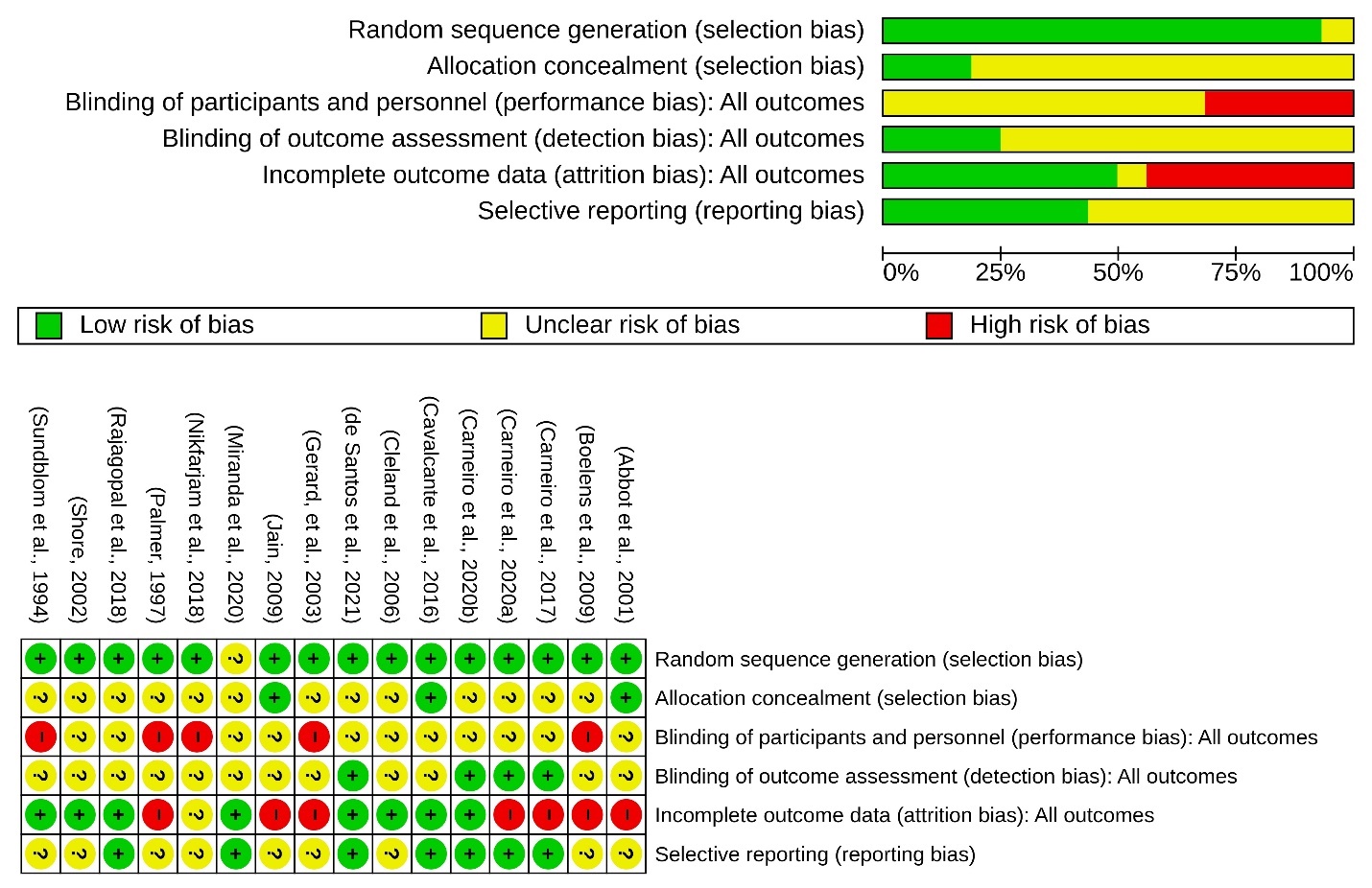


**Appendix E: Funnel Plots**


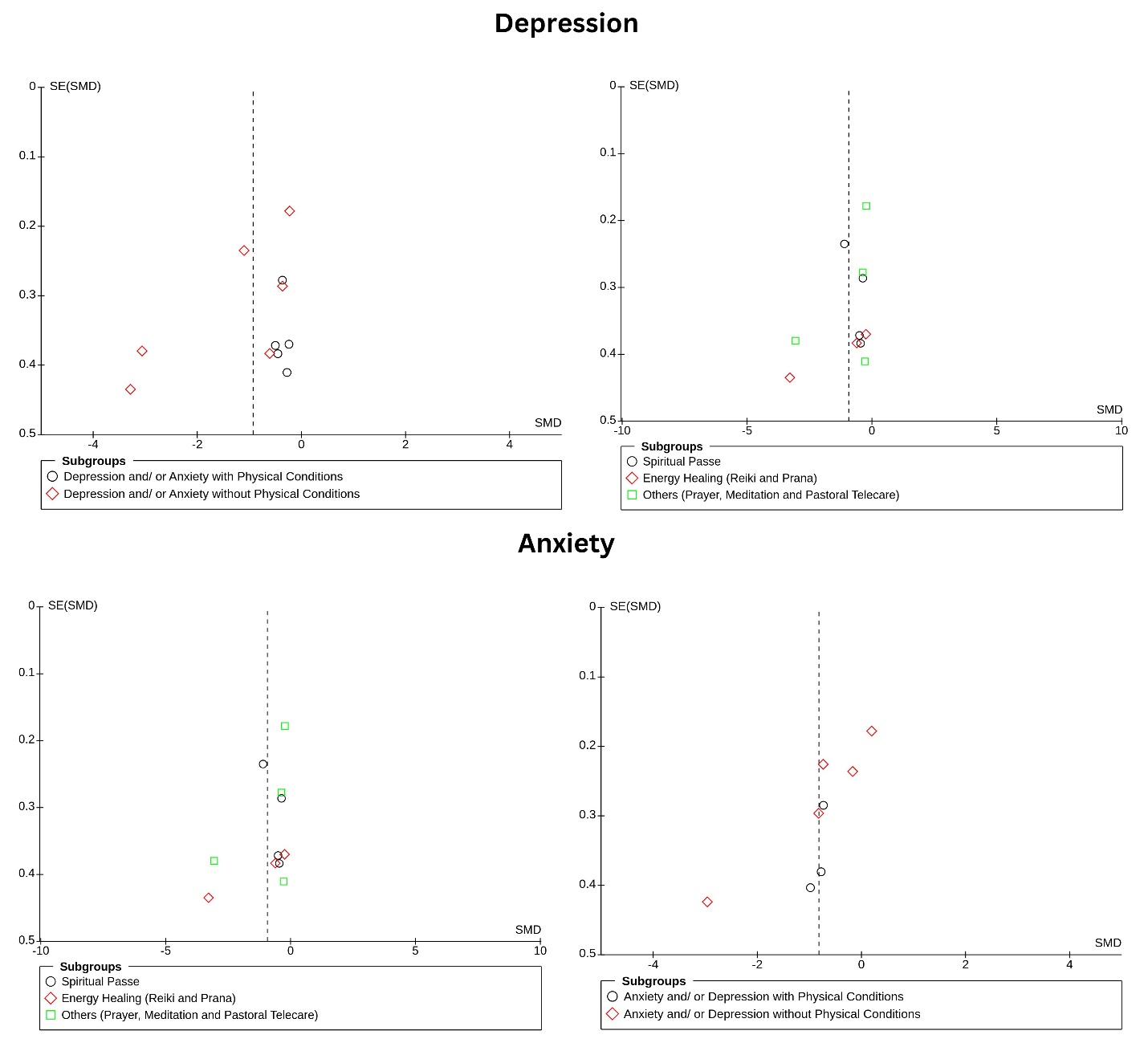


1. Traditional and Faith are defined as “healers who explicitly appeal to spiritual, magical, or religious explanations for disease and distress” [↑](#footnote-ref-1)
